# Investigating the relationships between concentrated disadvantage, place connectivity, and COVID-19 fatality in the United States over time

**DOI:** 10.1186/s12889-022-14779-1

**Published:** 2022-12-14

**Authors:** Fengrui Jing, Zhenlong Li, Shan Qiao, Jiajia Zhang, Bankole Olatosi, Xiaoming Li

**Affiliations:** 1 Department of Geography, Geoinformation and Big Data Research Lab, University of South Carolina, Columbia, SC 29208 USA; 2 Big Data Health Science Center, University of South Carolina, Columbia, SC 29208 USA; 3 Department of Health Promotion, Education, and Behavior, Arnold School of Public Health, University of South Carolina, Columbia, SC 29208 USA; 4 Department of Epidemiology and Biostatistics, Arnold School of Public Health, University of South Carolina, Columbia, SC 29208 USA; 5 Department of Health Services Policy and Management, Arnold School of Public Health, University of South Carolina, Columbia, SC 29208 USA

**Keywords:** COVID-19 fatality, Concentrated disadvantage, Twitter, Place connectivity, Moderation

## Abstract

**Background:**

Concentrated disadvantaged areas have been disproportionately affected by COVID-19 outbreak in the United States (US). Meanwhile, highly connected areas may contribute to higher human movement, leading to higher COVID-19 cases and deaths. This study examined the associations between concentrated disadvantage, place connectivity, and COVID-19 fatality in the US over time.

**Methods:**

Concentrated disadvantage was assessed based on the spatial concentration of residents with low socioeconomic status. Place connectivity was defined as the normalized number of shared Twitter users between the county and all other counties in the contiguous US in a year (*Y* = 2019). COVID-19 fatality was measured as the cumulative COVID-19 deaths divided by the cumulative COVID-19 cases. Using county-level (*N* = 3,091) COVID-19 fatality over four time periods (up to October 31, 2021), we performed mixed-effect negative binomial regressions to examine the association between concentrated disadvantage, place connectivity, and COVID-19 fatality, considering potential state-level variations. The moderation effects of county-level place connectivity and concentrated disadvantage were analyzed. Spatially lagged variables of COVID-19 fatality were added to the models to control for the effect of spatial autocorrelations in COVID-19 fatality.

**Results:**

Concentrated disadvantage was significantly associated with an increased COVID-19 fatality in four time periods (*p* < 0.01). More importantly, moderation analysis suggested that place connectivity significantly exacerbated the harmful effect of concentrated disadvantage on COVID-19 fatality in three periods (*p* < 0.01), and this significant moderation effect increased over time. The moderation effects were also significant when using place connectivity data from the previous year.

**Conclusions:**

Populations living in counties with both high concentrated disadvantage and high place connectivity may be at risk of a higher COVID-19 fatality. Greater COVID-19 fatality that occurs in concentrated disadvantaged counties may be partially due to higher human movement through place connectivity. In response to COVID-19 and other future infectious disease outbreaks, policymakers are encouraged to take advantage of historical disadvantage and place connectivity data in epidemic monitoring and surveillance of the disadvantaged areas that are highly connected, as well as targeting vulnerable populations and communities for additional intervention.

**Supplementary Information:**

The online version contains supplementary material available at 10.1186/s12889-022-14779-1.

## Introduction

Concentrated disadvantage, also known as neighborhood disadvantage, or deprivation index in some cases, refers to areas with a high proportion of people with low socioeconomic status. Concentrated disadvantaged areas aggregate groups such as low-income earners, welfare recipients, and single households [1], and some may also include ethnic minority groups [2]. These groups face a great number of challenges in socioeconomic development [2] and health wellbeing [3]. Due to social and cultural segregation, the disintegration of collective cohesion, limited institutional resources, dirty and disorderly environment, higher disadvantaged areas are associated with higher levels of crime rates [4] and fear of crime [5], antisocial behavior [6], intimate partner violence [7], violent victimization among youths [8], alcohol abuse [9], lower life satisfaction [10], adult unemployment and earnings [11], and negative educational outcomes [12]. Meanwhile, many studies have revealed the significant relationships between concentrated disadvantages and differential forms of health inequalities, such as the increased risk of breast cancer [13], increased incidence rate of lung cancer [14], diabetes and cholesterol control [15], obesity [16], pediatric obstructive sleep apnea [17], DNA methylation [18], adolescent brain cognitive development [19], depression [3, 20, 21], and worse mental health status [22].

Concentrated disadvantaged areas are more likely to suffer disproportionate COVID-19 infection [23] and deaths [24]. Some studies have also found a significant link between proxies of concentrated disadvantage (e.g., income) and case fatality rate (use fatality in the following section) [25]. The residents of concentrated areas are more likely to have poorer socioeconomic status and be essential workers in professions such as grocery delivery, truck drivers, and cleaners [26, 27]. Most of these jobs are difficult to perform remotely and lack the conditions to maintain social distancing. Meanwhile, disadvantaged populations may use public transportation more frequently, as a study in New York City found that areas with low-income people, essential workers, and non-white populations had more mobility extracted from subway data during the pandemic [28]. These groups also live in mostly poor house conditions, with many live together and without good post-infection isolation [29]. These factors of physical status, work environment, commuting patterns, and house conditions may contribute to a higher risk of exposure to COVID-19 and the increased likelihood of COVID-19 infection and fatality in socioeconomically disadvantaged populations.

Place connectivity is another key factor in predicting COVID-19 transmission among concentrated disadvantaged areas. The connectivity of a place can be described as the strength of a connection between a place and one or more places, and this connection is generally manifested in terms of the road, train, air, and social media, among others. Unlike direct population movements, connectivity is more stable, as it is closely related to geographical location, transportation facilities, and other related static factors. Place connectivity affects socioeconomic development and health outcomes of a region. Transportation connectivity (road, Internet, and air travel connectivity) improvements can promote economic growth by increasing market access and connecting intermodal terminals [30–32], as well as improve regional development by allowing different areas within the region to fully collaborate and reap the socio-economic benefits of integration [33, 34]. Greater accessibility is also associated with greater economic resilience in the region [35].

In terms of health effects, place connectivity can result in both positive and negative consequences. Transportation connectivity increases access to health care [32]. High transportation connectivity is also linked to lower levels of mental health distress [36]. Connectivity, on the other hand, is associated with some negative health outcomes, particularly infectious disease transmissions [37] (i.e., dengue outbreaks [38], influenza outbreaks [39], and HIV transmission [40]. For example, the intensity of air travel has been shown to be a significant predictor of virus arrival time [41]. The greater the connectivity between areas, the higher level of population mobility between these areas. Higher connectivity could be associated with a higher risk of exposure, and greater risk of COVID-19 infection. Several studies have found that air connectivity [37], high-speed train connectivity [42], road connectivity [43], and Twitter-based place connectivity [44] are associated with the initial outbreak of COVID-19. Particularly, Twitter-based connectivity, representing the extent to which a place shares the same users with other places, gives a comprehensive measure of the degree of connectivity in all aspects of transportation in that place, which can be a more direct proxy for population mobility and exposure risk [44].

There are a few studies with mixed results regarding the association between connectivity and COVID-19 clinical consequences (including fatality). Some studies have shown a significant association between air connectivity index and increased death [45] and death risk [46] in early-stage, while another suggested that pedestrian-oriented street connectivity is associated with lower COVID-19 death rates, because residents in this built environment engage in more physical activity and have lower levels of obesity and chronic disease [47].

Despite the above-mentioned studies, there are still knowledge gaps in investigating the relationships between concentrated disadvantage, place connectivity, and COVID-19 fatality. First, while several studies have investigated the effects of concentrated disadvantage and connectivity on COVID-19, most studies have focused on the incidence and mortality, with a paucity of studies linking concentrated disadvantage and COVID-19 fatality. As fatality is more influenced by pre-existing health conditions and the quality of the healthcare system [48], we hypothesize a significant association between concentrated disadvantage and COVID-19 fatality because concentrated disadvantage will be linked to health infrastructure in the area, access to health services, and pre-existing health conditions of a population on COVID-19 clinical outcomes.

Second, to the best of our knowledge, no study has yet evaluated the moderation effect of connectivity on the association between concentrated disadvantage and COVID-19 fatality. As many studies have confirmed [26, 49], people living in high concentrated disadvantaged areas may have higher needs to travel because most of them are essential workers and have limited resources to support remote working. In this case, if the area is also highly connected, these people may be more likely to take advantage of the convenient connectivity conditions (e.g., transportation) to go to work. Under the implementation of non-pharmacological interventions (NPIs) like travel restrictions during the pandemic, a high-connectivity place with a concentration of disadvantaged groups may have higher mobility compared to other high connectivity places without a concentration of disadvantaged groups. Higher mobility is associated with higher rates of infection [37, 42]. For people living in disadvantaged areas, a higher infection rate is usually linked with higher fatality given their poor pre-existing health conditions [50] and barriers to access to healthcare services [51]. Therefore, we hypothesize that connectivity may amplify the negative impacts of concentrated disadvantage on COVID-19 fatality.

Third, few studies investigate the associations between concentrated disadvantage, place connectivity, and COVID-19 outcomes across time [52, 53]. COVID-19 is constantly mutating and spreading, and the non-pharmaceutical COVID-19 prevention policies change over time. Place connectivity may not contribute to population movement in the same way at different periods, so the effect of place connectivity on concentrated disadvantage and COVID-19 fatality may vary. Travel restrictions were much stricter in the early period of the pandemic, resulting in decreased human mobility. In this situation, the impact of concentrated disadvantage on COVID-19 fatality may be less influenced by place connectivity. When life returns to normal and travel restrictions are lifted, such as during the Omicron variant period, the role of connectivity may become increasingly significant. As a result, we hypothesize that the moderation effect of place connectivity on the link of place connectivity – COVID-19 fatality varies along with the period of the pandemic.

In this paper, we use Twitter data to measure place connectivity. Twitter-based place connectivity is a comprehensive connectivity measurement among places, as previous studies have noted that it reflects connectivity not only in terms of transportation, but also in terms of social networks, geography, and socioeconomics [44]. Meanwhile, given the close association with these relatively static factors, place connectivity is a stable factor across years [44]. This study uses historical place connectivity to analyze its relationship with current COVID-19 fatality, which will be useful in guiding the role place connectivity may play in future infectious disease prevention and control.

In sum, this paper proposes that place connectivity can intensify the harmful effects of county-level concentrated disadvantage on county-level COVID-19 fatality. If a county with a concentration of disadvantaged populations is also a highly connected county, the disadvantaged group will have higher mobility through place connectivity and a greater probability of exposure to the virus, which may contribute to the deleterious effect of concentrated disadvantage on COVID-19 fatality. Our study will help to address the existing knowledge gaps and advance the understanding of complicated interaction between concentrated disadvantage, place connectivity, and COVID-19 fatality across pandemic periods. Specifically, we present the following hypotheses:

- H1: Concentrated disadvantage is associated with higher COVID-19 fatality.
- H2: The association between concentrated disadvantage and COVID-19 fatality is stronger in counties of high Twitter-based place connectivity compared to counties of low place connectivity.
- H3: The moderation effect of place connectivity may vary along with the period of the pandemic.

## Methods

### Data sources and study area

We obtained the county-level data for confirmed COVID-19 cases and deaths from the start of the outbreak on January 21, 2020, to December 1, 2022, in the contiguous US from the New York Times (https://github.com/nytimes/covid-19-data). This data was initially collected from the Center for Disease Control and Prevention (CDC), multilevel health departments, and other related sources. For data on county-level socioeconomic variables, we used the American Community Survey (ACS) 5-year estimates (2015-2019). Data from ACS 1-year or 3-year were not used because these data are limited to areas with populations over 20,000, and the current study intended to ensure data availability for smaller counties with populations less than 20,000 [26]. Twitter is one of the most popular social media platforms in the US and a very prevalent source of geospatial social media data in academia. We used place connectivity derived from Twitter in 2018 and 2019 [44].

The study area includes 3,091 of 3,108 counties (The District of Columbia is treated as a county equivalent) in the contiguous US. Omitted counties are due for two reasons. First, Twitter-based connectivity data covered 3,105 counties in the contiguous US. Second, since the subsequent empirical analysis used fatality for four time periods, we removed those counties with 0 cumulative COVID-19 cases in each time period, respectively. The spatial unit of this study is the county level.

### Measures

#### COVID-19 fatality

COVID-19 fatality is the outcome variable, which is the cumulative COVID-19 deaths divided by the cumulative COVID-19 cases up to a time period. The CDC evaluated key indicators for three periods of high COVID-19 transmission using data from three surveillance systems and a healthcare database [54]. According to the CDC report, there are three periods of high-COVID-19 transmission: December 1, 2020–February 28, 2021 (winter period); July 15, 2021–October 31, 2021 (Delta predominance); and December 19, 2021–January 15, 2022 (Omicron predominance). Correspondingly, the remaining were three normal-COVID-19 transmission periods. Because Omicron is less lethal and not quite the same as previous virus variants, we then selected data up to October 31, 2021, to test our hypotheses.

More specifically, this study includes models for four time periods, based on fatality data up to December 1, 2020 (period 1), up to February 28, 2021 (period 2), up to July 15, 2021 (period 3), and up to October 31, 2021 (period 4), respectively. We did not use single-period data (e.g., 12/1/2020-2/28/2021) to calculate fatality because the death population for a single period did not always belong to the cases in that single period.

#### Concentrated disadvantage

Data on concentrated disadvantage in each county were retrieved from the 5-year estimate American Community Survey (2015-2019). We first defined the concentrated disadvantage variable following previous studies [1, 26]. We then performed the principal component analysis of five variables and identified these variables loading onto a single factor that accounted for 58.24 % of the observed variation with high reliability (Cronbach’s Alpha α = 0.762). Concentrated disadvantages include five items, the civilian unemployment rate; the percentage of female-headed families; the percentage of the population over the age of 25 that are high school dropouts; the percentage of households with an annual income < $15,000; the percentage of households receiving public assistance. These items are combined into an index by taking the average of their z-scores. Higher values refer to a more concentrated disadvantage index.

#### Place connectivity

Place connectivity in this study is calculated based on place connectivity index (PCI) extracted through geotagged Tweets [44]. Unlike real-time population movement between places, PCI provides a relatively stable measure of the strength of connectivity between two places through spatial interaction. PCI refers to the normalized number of shared Twitter users between the two places in a year (Equation. 1). For instance, if a user is observed in both counties over a year, the user is considered a shared user in two counties. In this study, we aggregated the PCI values of a county with all other connected counties as the place connectivity of this county (Equation. 2). For instance, if county A has shared Twitter users with other 1,500 counties, that means county A has 1,500 PCI values. The place connectivity of county A is calculated by summing the 1,500 PCI values. Place connectivity is the moderator variable of this study. We calculated the place connectivity of each county separately for 2019 and 2018.

1 $$PCI_{ij}=\frac{S_{ij}}{S_iS_j}\;i,j\in\left[1,n\right]$$

2 $$Place\;connectivity_i=\sum\nolimits_i^nPCI_{in}\;i\in\left[1,n\right]$$

In the equations, ${S}_{i}$ is the number of unique Twitter users in county $i$ within time T; ${S}_{j}$ is the number of unique Twitter users in county $j$ within time T; ${S}_{ij}$ is the number of shared users between county $i$ and $j$ within time T; and $n$ is the number of counties in the study area.

#### Covariates

We also controlled for other main variables that may affect COVID-19 fatality. For socioeconomic aspects, population density, and uninsured population were included to account for their potential impacts [23]. For demographics, the percentage of the population aged 65 and older was included to control for high-risk groups with high COVID-19 fatality [23]. The percentage of black or African Americans was included to control for the impact of racial factors on COVID-19 [26]. In addition, the percentage of public transportation commuting was included to account for the impact of public transportation on COVID-19 transmission [55]; the percentage of ICU beds was also included to adjust for possible variation in COVID-19 deaths due to differences in availability of healthcare services [56]. For geographic factors, the geographical census region (Northeast, Midwest, South, and West) was to adjust for the potential impacts of environment-related factors (e.g., temperature and humidity) on the spread and severity of COVID-19 [4]. Core-based statistical area (CBSA) regions were included to adjust for the potential impact of urban and rural factors on COVID-19 fatality [57]. Just as some studies have used geographically weighted models to examine COVID-19 transmission and mortality to control for the role of spatially autocorrelated factors [23], this study incorporates spatially lagged fatality to ensure that the model can reduce the effect of spatially autocorrelated factors. Detailly, Moran’ *I* value of county-level COVID-19 fatality in the US were statistically significantly greater than 0 for different time periods, indicating that COVID-19 fatality was spatially correlated, we then included spatially lagged fatality (i.e., COVID-19 fatality in surrounding adjacent counties) to account for spatial autocorrelation of fatality. Table 1 summarized all the key variables in this study.

**Table 1:** Definitions of key variables

**Variables**	**Definitions**
***Outcome***	
COVID-19 fatality	The cumulative COVID-19 deaths divided by the cumulative COVID-19 cases up to a time period
***County-level predictors***	
*Demographic characteristics*	
Concentrated disadvantage	For counties with high percentages of residents of low socioeconomic status (welfare receipt, poverty, unemployment, uneducated, female-headed households)
Place connectivity	The total PCI value of the county
Spatially lagged fatality	The average fatality in the surrounding counties
Population density (per square miles)	The rate of total population to land area in the county
% of population aged 65 +	Proportion of population aged 65 + to total population
% of no health insurance coverage	Proportion of population with no health insurance coverage to total population
% of black or African Americans	Proportion of population of black or African descent to total population
% of workers 16 years and over who commute by public transportation	Proportion of population of workers 16 years and over who commute by public transportation
ICU bed per 100,000 people	The rate of ICU beds to total population in the county multiplied by 100,000
Core-based statistical area (CBSA)	
No-CBSA	0
Micropolitan statistical area	1
Metropolitan statistical area	2
Region	
Northeast	0
Midwest	1
South	2
West	3

### Statistical analysis

The count data for COVID-19 deaths were highly right-skewed and overdispersed. As Poisson regression could not capture overdispersion, the negative binomial model is more appropriate. Considering the differential impact of COVID-19 fatality rates by state-level policies such as social distance, face masks, and home orders, we further selected a mixed-effects negative binomial regression model to account for state-level random effects on COVID-19 fatality at the county level [26]. In each model, to calculate the fatality, the number of COVID-19 deaths was the dependent variable, and the number of COVID-19 cases was the offset term. To avoid numerical singularities in estimating the models, we log-transformed certain variables (population density, place connectivity, and spatially lagged fatality) to ensure accurate analytical results. Before the regression analysis, we used Pearson correlation and VIF analysis to examine possible co-collinearity. The results show that no significant co-collinearity exists between variables (VIF values less than 4). We performed statistical analyses in Stata SE version 15.

## Results

### Descriptive statistics and spatial characteristics

Table 2 presents the descriptive statistics of the variables. By October 31, 2021 (period 4), the county-level average fatality is 0.018. The score range for concentrated disadvantage was from -1.442 to 4.916, with a mean value of -0.073. The average place connectivity in 2019 was 5,634.516, varying from 1,167.885 to 24,065.32. The statistics of other variables were shown in Table 2.

**Table 2:** The descriptive statistics of the variables

	Mean	SD	Min	Max
Fatality in time period 1	0.02	0.018	0	0.196
Fatality in time period 2	0.019	0.011	0	0.111
Fatality in time period 3	0.02	0.01	0	0.111
Fatality in time period 4	0.018	0.008	0	0.075
Concentrated disadvantage	-0.073	0.592	-1.442	4.916
Place connectivity 2019	5,634.516	2,884.957	1,167.885	24,065.32
Place connectivity 2018	6,048.58	3,164.618	1,154.232	26,156.29
Spatial lagged fatality in time period 1	0.02	0.011	0.001	0.094
Spatial lagged fatality in time period 2	0.019	0.007	0.004	0.053
Spatial lagged fatality in time period 3	0.02	0.006	0.004	0.052
Spatial lagged fatality in time period 4	0.018	0.005	0.004	0.045
population density	219.266	811.391	0.207	18,654.76
% of population aged 65 +	18.835	4.591	3.2	56.71
% of no health insurance	9.558	4.972	0.67	40.91
% of black or African population	9.164	14.579	0	87.23
% of workers 16 years and over who commute by public transportation	0.891	2.321	0	43.3
ICU beds per 100,000 population	12.686	23.591	0	749.584
	Frequency	Percentage		
CBSA (Non-CBSA)	701	22.68		
Micro	854	27.63		
Metro	1,536	49.69		
Region (Northeast)	210	6.79		
Midwest	1049	33.94		
South	1,421	45.97		
West	411	13.3		

**Fig. 1:**
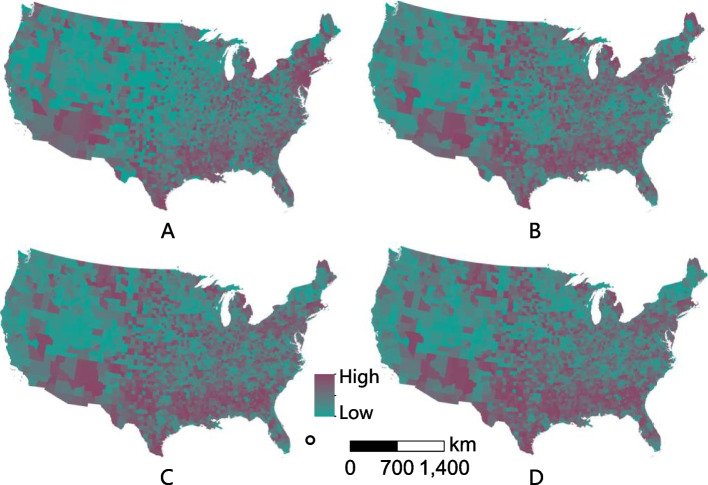
County-level COVID-19 fatality across the contiguous US. Note: The figures on the left side from A to D show the distribution of fatality for period 1, period 2, period 3, and period 4, respectively

The geospatial distribution of fatality in the contiguous US shows that the high fatality area was widely distributed and tends to be concentrated in the South (Figure 1). Economically developed regions like California did not show an excessive fatality. From the four time periods, the areas of high fatality changed over time. Initially, hotspots were in the Northeast and Southwest, and then gradually spread to the interior and surrounding regions. This may be related to coronavirus transmission, as the outbreak first occurred in the metropolitan areas of the east and west coasts.

**Fig. 2:**
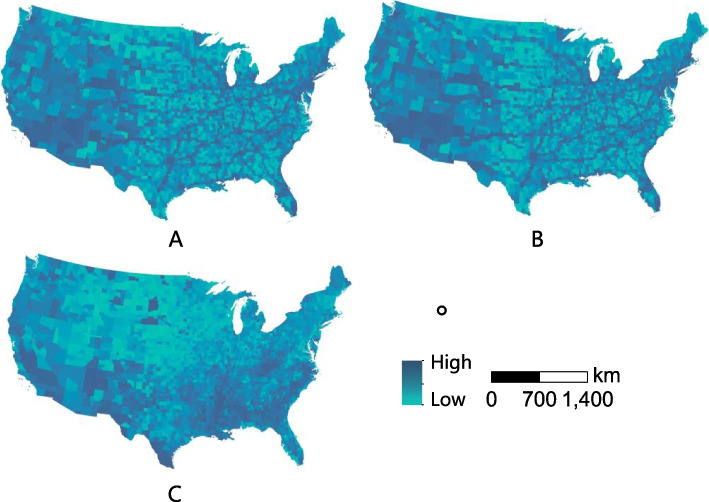
Twitter-based place connectivity across the contiguous US. Note: A and B refer to place connectivity for 2019 and 2018, separately. C refers to the map of concentrated disadvantage

Figure 2 showed the geospatial distribution of Twitter-based place connectivity and concentrated disadvantage. It can be observed that high connected counties were like those major transportation nodes. The Northeast and Southwest were areas of higher place connectivity, which contained some notable metropolitan areas, such as San Francisco, Los Angeles, and New York City. The spatial distribution of place connectivity was similar in 2019 and 2018. The map of concentrated disadvantage showed that high disadvantaged counties were mainly located in the South, while coastal areas and some northern areas showed lower levels of disadvantage (Figure 2).

### The results of mixed-effects negative binomial regression models

The results used the incidence rate ratio (IRR) to represent the association between the variables. An IRR greater than 1 represents a positive association between county-level factors and COVID-19 fatality. In contrast, an IRR less than 1 represents a negative association, and an IRR equal to 1 represents no positive or negative association. Model 1 in Tables 3 and 4 showed the results of mixed-effect negative binomial regression analysis up to the four-time points. In each period, counties with higher disadvantages had higher fatality than those with lower disadvantages (*IRR* > 1, *p* < 0.01). For example, in period 4, compared to counties with lower concentrated disadvantage, the fatality in counties with higher concentrated disadvantage was 1.157 times higher. (*IRR* = 1.157, 95% *CI*: 1.125-1.189, *p* < 0.01). It indicates hypothesis 1 is confirmed. The effects of place connectivity were significant in period 2, 3, & 4 (*IRR* <1, *p* < 0.01), but not in period 1(*IRR* < 1, *p* > 0.05). Specifically, counties with higher place connectivity had lower fatality than counties with lower place connectivity.

**Table 3:** Mixed-effects negative binomial regression models of county-level COVID-19 fatality (periods 1 & 2)

	Period 1		Period 2	
Factors	Model 1	Model 2	Model 1	Model 2
	IRR (95% *CI*)	IRR (95% *CI*)	IRR (95% *CI*)	IRR (95% *CI*)
Concentrated disadvantage	1.185(1.119,1.255) **	1.188(1.121, 1.258) **	1.156(1.117,1.197) **	1.166(1.126,1.206) **
Log (PC)	0.93(0.812,1.065)	0.908(0.791, 1.044)	0.878(0.812,0.951) **	0.852(0.786,0.923) **
Concentrated disadvantage * Log (PC)		1.142(0.958, 1.361)		1.267(1.141,1.406) **
Log (Spatially lagged fatality)	2.519(2.2,2.884) **	2.526(2.207, 2.892) **	2.799(2.467,3.176) **	2.795(2.464,3.169) **
Log (population density)	1.089(1.03,1.152) **	1.094(1.034, 1.157) **	1.026(0.992,1.06)	1.031(0.997,1.066)
% of population aged 65 +	1.039(1.033,1.046) **	1.039(1.033, 1.045) **	1.035(1.032,1.039) **	1.035(1.031,1.038) **
% of no health insurance	1(0.993,1.007)	1.000(0.993, 1.007)	1.008(1.004,1.013) **	1.008(1.004,1.012) **
% of black or African Americans	1.004(1.002,1.006) **	1.004(1.002, 1.006) **	1.002(1,1.003) **	1.002(1,1.003) *
% of workers 16 years and over who commute by public transportation	1.008(0.998,1.018)	1.008(0.998, 1.018)	1.005(0.999,1.011)	1.005(0.999,1.011)
ICU beds per 100,000 population	1(0.999,1.001)	1(0.999, 1.001)	1(1,1.001)	1(1,1.001)
CBSA (Non-CBSA)				
Micro	0.963(0.9,1.03)	0.961(0.898, 1.028)	0.961(0.924,1) *	0.958(0.921,0.996) *
Metro	0.968(0.906,1.034)	0.965(0.903, 1.031)	0.95(0.914,0.987) **	0.946(0.91,0.983) **
Region (Northeast)				
Midwest	0.616(0.511,0.742) **	0.614(0.510, 0.740) **	0.889(0.788,1.003)	0.884(0.784,0.997) *
South	0.644(0.539,0.769) **	0.645(0.540, 0.770) **	0.822(0.731,0.923) **	0.824(0.734,0.925) **
West	0.62(0.507,0.758) **	0.619(0.507, 0.757) **	0.792(0.696,0.901) **	0.79(0.694,0.899) **

*IRR* Incidence rate ratio, *PC* Place connectivity 2019, *CI* Confidence interval

^*^: *p* < 0.05

^**^: *p* < 0.001

**Table 4:** Mixed-effects negative binomial regression models of county-level COVID-19 fatality (periods 3 & 4)

	Period 3		Period 4	
Factors	Model 1	Model 2	Model 1	Model 2
	IRR (95% *CI*)	IRR (95% *CI*)	IRR (95% *CI*)	IRR (95% *CI*)
Concentrated disadvantage	1.172(1.137,1.208) **	1.182(1.147,1.219) **	1.157(1.125,1.189) **	1.167(1.135,1.199) **
Log (PC)	0.885(0.826,0.948) **	0.854(0.796,0.916) **	0.923(0.867,0.983) *	0.888(0.834,0.946) **
Concentrated disadvantage * Log (PC)		1.303(1.189,1.428) **		1.321(1.216,1.436) **
Log (Spatially lagged fatality)	2.766(2.449,3.124) **	2.758(2.444,3.113) **	2.909(2.578,3.283) **	2.892(2.566,3.26) **
Log (population density)	1.016(0.987,1.046)	1.022(0.992,1.052)	1.009(0.983,1.036)	1.015(0.989,1.042)
% of population aged 65 +	1.035(1.032,1.039) **	1.034(1.031,1.038) **	1.033(1.03,1.036) **	1.032(1.029,1.035) **
% of no health insurance	1.008(1.004,1.012) **	1.008(1.004,1.011) **	1.008(1.004,1.011) **	1.008(1.004,1.011) **
% of black or African Americans	1.002(1.001,1.003) **	1.002(1.001,1.003) **	1.002(1.001,1.003) **	1.002(1.001,1.003) **
% of workers 16 years and over who commute by public transportation	1.004(0.999,1.009)	1.004(0.999,1.009)	1.003(0.999,1.008)	1.003(0.999,1.008)
ICU beds per 100,000 population	1(1,1.001)	1(1,1.001)	1(1,1.001)	1(1,1.001)
CBSA (Non-CBSA)				
Micro	0.958(0.926,0.992) **	0.954(0.922,0.988) **	0.973(0.943,1.004)	0.969(0.94,1) *
Metro	0.957(0.925,0.99) **	0.952(0.921,0.985) **	0.968(0.939,0.998) *	0.963(0.935,0.993) *
Region (Northeast)				
Midwest	0.974(0.867,1.094)	0.968(0.862,1.087)	0.978(0.876,1.092)	0.973(0.872,1.086)
South	0.924(0.826,1.034)	0.928(0.83,1.037)	0.951(0.856,1.058)	0.955(0.859,1.062)
West	0.861(0.76,0.974) *	0.859(0.759,0.971) *	0.901(0.802,1.012)	0.899(0.801,1.009)

*IRR* Incidence rate ratio, *PC* Place connectivity 2019, *CI* Confidence interval

^*^: *p* < 0.05

^**^: *p* < 0.001

**Fig. 3:**
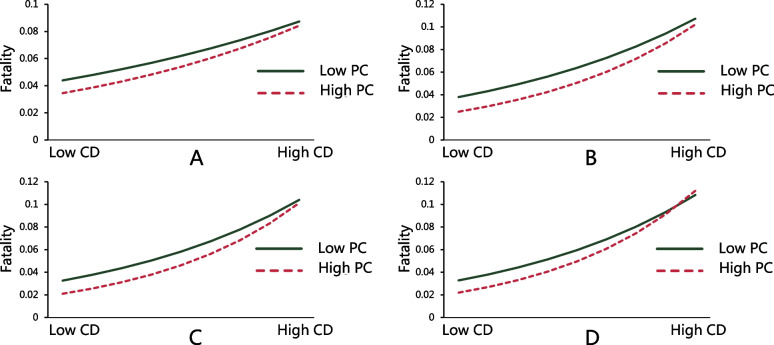
The impacts of concentrated disadvantage on COVID-19 fatality by different place connectivity. Note: figures A to D refer to the models for time periods 1 to 4, respectively (the interaction for period 1 is not significant). CD refers to concentrated disadvantage, and PC refers to place connectivity

Further, the results showed that the interaction between concentrated disadvantage and place connectivity was significant in three time periods (*IRR >* 1, *p* < 0.01), except for period 1(*IRR* > 1, *p* > 0.05), after the inclusion of an interaction term (Model 2, Tables 3 and 4). It supports hypothesis 2, implying that concentrated disadvantage is associated with a relative increase in the county-level COVID-19 fatality, especially for those counties with high place connectivity. Interestingly, the IRR of this interaction increased with the time periods, indicating an increasing robust interaction effect (Tables 3 and 4 and Figure 3), which confirms hypothesis 3. Figure 3 shows the graphical illustration of the interaction between concentrated disadvantage and place connectivity using results from Model 2, Tables 3 and 4. The relationship between concentrated disadvantage and fatality becomes stronger as the value of place connectivity increases.

Most control variables also showed consistent results across time (Model 1, Tables 3 and 4). Each 1 standard deviation increase in spatial lagged fatality rate was associated with a significant relative increase in the IRR of COVID-19 fatality (*IRR* > 1 and *p* < 0.01). A higher percentage of people aged 65 and older was associated with a higher COVID-19 fatality (*IRR* > 1 and *p* < 0.01). Similarly, a higher percentage of Black or African Americans was associated with a higher COVID-19 fatality (*IRR* > 1 and *p* < 0.01). In addition, except for period 1, micro and metropolitan counties had lower COVID-19 fatality than rural counties (*IRR* < 1 and *p* < 0.01). The region factor was not consistently significant. Over time, the significant regional differences in COVID-19 fatality gradually disappeared. The remaining control variables did not show significant results.

We also ran the models using the 2018 place connectivity data (Tables S1 to S2 in Supplementary Material), which showed consistent results to the model using 2019 place connectivity data.

## Discussion

Leveraging concentrated disadvantage and Twitter-based place connectivity, we examined the relationship between concentrated disadvantage and COVID-19 fatality in the US, and how this association is moderated by place connectivity. In addition to examining the harmful effect of concentrated disadvantage, this study partially explored the mechanism of this effect. The significant interaction between place connectivity and concentrated disadvantage suggests that socioeconomically disadvantaged groups in an area with high levels of place connectivity may be more likely to experience higher mobility, and thus face higher incidence and fatality risk. The results provide new insights into the association between concentrated disadvantage and COVID-19 fatality and may provide some guidance for future infectious disease control policies in socioeconomically disadvantaged areas.

We further found that the moderation effect of place connectivity increased over time, which may be related to increased mobility and the loosening of travel restrictions. At the early stages of the pandemic, COVID-19 fatality was more severe, travel restrictions were higher, and people were also in a precautionary awareness to reduce their outside activities, so the effect of concentrated disadvantage on fatality may be less influenced by place connectivity. In contrast, with widespread vaccination, life returns to normal and daily travel is less restricted, thus the moderation effect of place connectivity on the link between concentrated disadvantage and COVID-19 fatality became increasingly significant.

The significant association between place connectivity and decreased COVID-19 fatality rate is observed in time periods 2 to 4. We found a moderate correlation between population density, ICU percentage, and place connectivity. The high fatality was mostly found in districts with low population density due to poorer health care systems [58]. Rural areas hold less access to health facilities, but urban areas, which are more likely to encounter large numbers of cases, instead have better health facility preparation and prevention to avoid more deaths [57]. It implies that highly connected areas are generally areas with higher population density and urbanization, and may have better medical conditions and facilities, leading to a lower fatality. This finding is consistent with associations between urbanization [59] and population density [58], and lower COVID-19 fatality. However, the effect of place connectivity on COVID-19 was not significant in period 1, probably due to strict travel restrictions and low travel needs in the early stage, resulting in connectivity not working.

Our findings have public health implications in the practice of responding to infectious disease epidemics/pandemics in terms of disease surveillance and monitoring as well as resource allocation and health equalities. Timely monitoring of outbreaks in concentrated disadvantaged areas with high place connectivity can aid in identifying potential epidemic hotspots and vulnerable areas, which will contribute to evidence-based decision-making in secondary prevention strategies and efforts. In addition, our study results suggest the importance of resource allocation measures favoring the areas with high socioeconomic disadvantage and high place connectivity. For example, financial assistance integrated into the transportation restriction policy can significantly reduce the mobility of concentrated disadvantaged neighborhoods. Vaccination promotion via free supplements and increased vaccine administration sites among the vulnerable population will improve community immunity toward the virus. These strategies reduce disproportionate COVID-19 fatality, interrupt transmission, and improve health equities among concentrated disadvantaged areas.

There are a few limitations to this study. First, place connectivity is measured from Twitter data, while is less used by some groups, such as the elderly and children. Also, data in some counties where Twitter is less used may be underrepresented. Second, low-income populations may be less likely to report their illness when the symptoms are mild. Similarly, disadvantaged populations may be less likely to take COVID-19 testing at all due to the access barriers to relevant healthcare services. These will result in underestimating COVID-19 cases and biasing the fatality. Third, utilizing county-level data to understand the effects of concentrated disadvantage on COVID-19 fatality may ignore the role of neighborhood-level factors. There may exist several neighborhoods with high socioeconomic status even in concentrated disadvantaged counties. Last, this study used spatial-scale variables rather than individual-level COVID-19 data, hence the results can only indicate the associations between geospatial environment and COVID-19 outcomes and cannot be interpreted as individual-level associations or causalities. Future multilevel analyses could be applied, including data on individual characteristics and neighborhood factors, which could yield more robust findings.

## Conclusion

Concentrated disadvantage contributes to the geospatial disparities in county-level COVID-19 fatality in the US: counties with higher levels of socioeconomic disadvantage reported higher levels of COVID-19 fatality. Place connectivity moderates the detrimental effects of concentrated disadvantage on fatality, and this moderation effect increases along with time periods. Our findings not only further explain the link between concentrated disadvantage and COVID-19 fatality, but also further highlights the role of place connectivity in combating COVID-19 and future infectious diseases.

Practically, more policies should be implemented to concentrated disadvantaged counties to reduce disproportionate COVID-19 fatality in these areas. Particularly for counties with high socioeconomic disadvantage and high place connectivity, policies such as more financial assistance (to radically reduce the mobility of the poor) and vaccination supplement (to reduce the physical vulnerability of the poor) should be considered to reduce more disproportionate deaths in these areas. Timely monitoring of outbreaks in concentrated disadvantaged areas with high place connectivity can aid in identifying potential epidemic hotspots and vulnerable areas, which can contribute to evidence-based decision-making in resource allocation to combat the pandemic and potentially other emerging infectious diseases in the future.

## Supplementary Information

**Additional file 1: Table S1. **Mixed-effects negative binomialregression models of county-level COVID-19 fatality (periods 1 & 2). **Table S2.** Mixed-effects negative binomialregression models of county-level COVID-19 fatality (periods 3 & 4).

## Data Availability

The datasets used and/or analyzed during the current study are available in the New York Times GitHub repository (https://github.com/nytimes/COVID19-data), the United States Census Bureau (https://www.census.gov/programs-surveys/acs/data.html), and the Geoinformation and Big Data Research Laboratory at University of South Carolina (http://gis.cas.sc.edu/gibd/place-connectivity-index/). Additional datasets used and/or analysed during the current study are available from the corresponding author on reasonable request.
