# Supplementary material for: Investigating the relationships between concentrated disadvantage, place connectivity, and COVID-19 fatality in the United States over time
Source: BMC Public Health. 2022 Dec 14;22:2346. doi: 10.1186/s12889-022-14779-1 (PMC9748905; doi:10.1186/s12889-022-14779-1)
Supplement: Supplementary file 1 — Additional file 1: Table S1. Mixed-effects negative binomialregression models of county-level COVID-19 fatality (periods 1 & 2). Table S2. Mixed-effects negative binomialregression models of county-level COVID-19 fatality (periods 3 & 4). [file 12889_2022_14779_MOESM1_ESM.docx]

# **Table S1**

Table S1. Mixed-effects negative binomial regression models of county-level COVID-19 fatality (periods 1 & 2)

|  | Period 1 | | Period 2 | |
| --- | --- | --- | --- | --- |
| Factors | Model 1 | Model 2 | Model 1 | Model 2 |
|  | IRR (95% *CI*) | IRR (95% *CI*) | IRR (95% *CI*) | IRR (95% *CI*) |
| Concentrated disadvantage | 1.187(1.121,1.258) ** | 1.188(1.121,1.258) ** | 1.158(1.119,1.199) ** | 1.163(1.123,1.203) ** |
| Log (PC) | 0.965(0.844,1.103) | 0.96(0.836,1.101) | 0.917(0.847,0.992) * | 0.898(0.83,0.973) ** |
| Concentrated disadvantage * Log (PC) |  | 1.032(0.865,1.232) |  | 1.171(1.054,1.301) ** |
| Log (Spatially lagged fatality) | 2.521(2.201,2.886) ** | 2.523(2.203,2.889) ** | 2.807(2.473,3.186) ** | 2.806(2.473,3.184) ** |
| Log (population density) | 1.083(1.024,1.145) ** | 1.084(1.024,1.147) ** | 1.019(0.986,1.054) | 1.023(0.989,1.058) |
| % of population aged 65 + | 1.04(1.033,1.046) ** | 1.04(1.033,1.046) ** | 1.036(1.032,1.039) ** | 1.035(1.031,1.039) ** |
| % of no health insurance | 1(0.993,1.007) | 1(0.993,1.007) | 1.008(1.004,1.013) ** | 1.008(1.004,1.013) ** |
| % of black or African Americans | 1.004(1.002,1.006) ** | 1.004(1.002,1.006) ** | 1.002(1,1.003) ** | 1.002(1,1.003) * |
| % of workers 16 years and over who commute by public transportation | 1.008(0.998,1.018) | 1.008(0.998,1.018) | 1.005(0.999,1.011) | 1.005(0.999,1.011) |
| ICU beds per 100,000 population | 1(0.999,1.001) | 1(0.999,1.001) | 1(1,1.001) | 1(1,1.001) |
| CBSA (Non-CBSA) |  |  |  |  |
| Micro | 0.963(0.9,1.03) | 0.963(0.9,1.03) | 0.962(0.925,1) | 0.96(0.923,0.999) * |
| Metro | 0.967(0.905,1.034) | 0.967(0.905,1.033) | 0.95(0.914,0.988) * | 0.948(0.912,0.986) ** |
| Region (Northeast) |  |  |  |  |
| Midwest | 0.616(0.511,0.742) ** | 0.616(0.511,0.742) ** | 0.89(0.789,1.004) | 0.887(0.787,1) * |
| South | 0.644(0.539,0.769) ** | 0.644(0.539,0.769) ** | 0.821(0.731,0.922) ** | 0.824(0.734,0.925) ** |
| West | 0.616(0.503,0.754) ** | 0.616(0.504,0.754) ** | 0.787(0.692,0.896) ** | 0.787(0.691,0.895) ** |

*Notes*: IRR: incidence rate ratio; PC: place connectivity 2018; *CI*: Confidence interval; *: *p* < 0.05; **: *p* < 0.001.

# **Table S2**

Table S2. Mixed-effects negative binomial regression models of county-level COVID-19 fatality (periods 3 & 4)

|  | Period 3 | | Period 4 | |
| --- | --- | --- | --- | --- |
| Factors | Model 1 | Model 2 | Model 1 | Model 2 |
|  | IRR (95% *CI*) | IRR (95% *CI*) | IRR (95% *CI*) | IRR (95% *CI*) |
| Concentrated disadvantage | 1.173(1.138,1.209) ** | 1.179(1.143,1.215) ** | 1.158(1.126,1.19) * | 1.164(1.132,1.196) ** |
| Log (PC) | 0.902(0.842,0.966) ** | 0.877(0.818,0.94) ** | 0.942(0.885,1.002) | 0.913(0.858,0.972) ** |
| Concentrated disadvantage * Log (PC) |  | 1.238(1.129,1.358) ** |  | 1.248(1.148,1.357) ** |
| Log (Spatially lagged fatality) | 2.768(2.45,3.126) ** | 2.762(2.447,3.118) ** | 2.908(2.577,3.282) ** | 2.889(2.562,3.258) ** |
| Log (population density) | 1.014(0.984,1.044) | 1.019(0.989,1.049) | 1.006(0.98,1.033) | 1.011(0.985,1.039) |
| % of population aged 65 + | 1.035(1.032,1.039) ** | 1.035(1.031,1.038) ** | 1.033(1.03,1.036) ** | 1.032(1.029,1.035) ** |
| % of no health insurance | 1.008(1.004,1.012) ** | 1.008(1.004,1.012) ** | 1.008(1.005,1.012) ** | 1.008(1.004,1.011) ** |
| % of black or African Americans | 1.002(1.001,1.003) ** | 1.002(1.001,1.003) ** | 1.002(1.001,1.003) ** | 1.002(1.001,1.003) ** |
| % of workers 16 years and over who commute by public transportation | 1.004(0.999,1.009) | 1.004(0.999,1.009) | 1.003(0.999,1.008) | 1.003(0.998,1.008) |
| ICU beds per 100,000 population | 1(1,1.001) | 1(1,1.001) | 1(1,1.001) | 1(1,1.001) |
| CBSA (Non-CBSA) |  |  |  |  |
| Micro | 0.959(0.927,0.993) ** | 0.957(0.925,0.99) * | 0.974(0.944,1.004) | 0.971(0.942,1.002) |
| Metro | 0.958(0.926,0.991) ** | 0.955(0.923,0.988) ** | 0.969(0.94,0.999) * | 0.966(0.937,0.995) * |
| Region (Northeast) |  |  |  |  |
| Midwest | 0.975(0.868,1.096) | 0.971(0.865,1.09) | 0.979(0.877,1.093) | 0.975(0.873,1.088) |
| South | 0.924(0.826,1.034) | 0.928(0.83,1.037) | 0.951(0.855,1.057) | 0.956(0.86,1.062) |
| West | 0.859(0.759,0.973) * | 0.858(0.759,0.971) * | 0.899(0.8,1.009) | 0.898(0.8,1.008) |

*Notes*: IRR: incidence rate ratio; PC: place connectivity 2018; *CI*: Confidence interval; *: *p* < 0.05; **: *p* < 0.001.
